# Hormonal drugs: Influence on growth, biofilm formation, and adherence of selected gut microbiota

**DOI:** 10.3389/fcimb.2023.1147585

**Published:** 2023-03-13

**Authors:** Zainab K. Hammouda, Reham Wasfi, Nourtan F. Abdeltawab

**Affiliations:** ^1^ Department of Microbiology and Immunology, Faculty of Pharmacy, October University for Modern Sciences and Arts (MSA), Giza, Egypt; ^2^ Department of Microbiology and Immunology, Faculty of Pharmacy, Cairo University, Cairo, Egypt

**Keywords:** gut microbiota, hormones, *Bacteroides fragilis*, *Bifidobacterium longum*, *Escherichia coli*, *Limosilactobacillus reuteri*, short-chain fatty acids (SCFAs)

## Abstract

Many studies have reported the influence of hormonal drugs on gut microbiota composition. However, the underlying mechanism of this interaction is still under study. Therefore, this study aimed to evaluate the possible *in vitro* changes in selected members of gut bacteria exposed to oral hormonal drugs used for years. Selected members of gut bacteria were *Bifidobacterium longum, Limosilactobacillus reuteri*, *Bacteroides fragilis, and Escherichia coli* representing the four main phyla in the gut. Selected hormonal drugs used for a long time were estradiol, progesterone, and thyroxine. The effect of intestinal concentrations of these drugs on the selected bacterial growth, biofilm formation, and adherence to Caco-2/HT-29 cell line was assessed. Short-chain fatty acids (SCFAs) have been included in host functions including the gut, immune and nervous functions; thus, the drug’s effects on their production were assayed using High- Performance Liquid Chromatography. Sex steroids significantly increased the growth of all tested bacteria except *B. longum*, similarly, thyroxine increased the growth of tested Gram-negative bacteria however reducing that of tested Gram-positive bacteria. The effect of drugs on biofilm formation and bacterial adherence to cell lines cocultures was variable. Progesterone decreased the biofilm formation of tested Gram-positive bacteria, it nevertheless increased *L. reuteri* adherence to Caco-2/HT-29 cell line cell lines coculture. By contrast, progesterone increased biofilm formation by Gram-negative bacteria and increased adherence of *B. fragilis* to the cell lines coculture. Moreover, thyroxine and estradiol exhibited antibiofilm activity against *L. reuteri*, while thyroxine increased the ability of *E. coli* to form a biofilm. Moreover, hormones affected bacterial adherence to cell lines independently of their effect on hydrophobicity suggesting other specific binding factors might contribute to this effect. Tested drugs affected SCFAs production variably, mostly independent of their effect on bacterial growth. In conclusion, our results showed that the microbiota signature associated with some hormonal drug consumption could be the result of the direct effect of these drugs on bacterial growth, and adherence to enterocytes besides the effect of these drugs on the host tissue targets. Additionally, these drugs affect the production of SCFAs which could contribute to some of the side effects of these drugs.

## 1 Introduction

Gut bacteria play an important role in maintaining human health through metabolic, protective, and trophic mechanisms; however, altering their composition has been linked to the development of certain diseases such as irritable bowel disease (IBD), colorectal cancer, obesity, diabetes, autism, etc. (Prakash et al., 2011; DeGruttola et al., 2016). Among the important functions played by the gut bacteria is the production of short-chain fatty acids (SCFAs) as the end products of the metabolism of complex carbohydrates. SCFAs have several effects on maintaining the health and integrity of the human body besides being an energy source for colonocytes, having a strong anti-inflammatory effect, maintaining the integrity of the blood-brain barrier (BBB), and reducing the oxidative stress in the colon. The most abundant SCFAs produced in the colon are acetate, butyrate, and propionate. (Prakash et al., 2011; Valdes-Varela et al., 2016; Silva et al., 2020). 

Different types of microorganisms, including bacteria, yeast, and viruses, make up the gut microbiota. *Bacteroidetes* and *Firmicutes* account for 90% of the intestinal bacteria, and *Proteobacteria, Actinobacteria*, and *Verrucomicrobia* comprise the other major phyla that constitute the gut (Scotti et al., 2017; Rinninella et al., 2019). Microbiota composition and functions are exposed to dysbiosis by host factors and environmental pressure (Thursby and Juge, 2017). Among these factors contributing to possible dysbiosis is drug intake (Nicholson et al., 2012; Hasan and Yang, 2019).

Research in population-based cohorts has revealed a connection between various non-antibiotic drug classes and specific gut microbiome signatures highlighting that this interaction might contribute to the development of diseases in addition to the change in drug metabolism by gut microbiota (Vich Vila et al., 2020). Among the drugs that have drawn the attention of researchers to study their effect on gut microbiota are hormonal drugs because they are usually used for a long time ranging from 3 months to a lifetime (Benvenga et al., 2017; Clabaut et al., 2021; Gong et al., 2021). However, the direct effect of these drugs on the growth, adherence, and production of SCFAs by gut bacteria was not studied *in vitro* except for a few studies that utilized concentrations higher than the estimated intestinal concentrations. Therefore, this study aimed to study the impact of hormonal drugs at the intestinal concentration on the growth, adherence, and production of SCFAs and lactic acid by selected members of the gut microbiota to explain the *in vivo* changes in gut microbiota upon consumption of hormonal drugs.

## 2 Materials and methods

### 2.1 Bacterial strains and culturing condition

Four bacterial strains were used including *Bacteroides fragilis* (ATCC 25285), *Bifidobacterium longum* (ATCC 15707), *Escherichia coli* (ATCC 8739), and *Limosilactobacillus reuteri* (ATCC 23272) representing the 4 main phyla *Bacteroidetes, Actinobacteria, Proteobacteria*, and *Firmicutes*, respectively, which comprise the gut microbiota.

All strains were cultured in their recommended media: De Man, Rogosa & Sharpe (MRS) broth and lactobacillus selective (LBS) agar for *L. reuteri*; Brain-heart infusion supplemented (BHIS) broth and neomycin anaerobic blood agar (NABA) for *Bacteroides fragilis*; Reinforced clostridial media (RCM) broth and MRS agar supplemented with 0.05% cysteine for *Bifidobacterium longum*; Luria Bertani (LB) broth and MacConkey agar for *E. coli*. All the previous cultures were incubated under anaerobic conditions (BBL anaerobic jar, Gas pack Anaerobic system, Franklin, New Jersey, USA) at 37°C. Strains were preserved in glycerol stock at −20°C.

### 2.2 Preparation of drug stock solutions

Steroid sex hormones of ethinyl estradiol and progesterone were obtained from Qinhuangdao Zizhu Pharmaceutical (Hebei, China) and Zhejiang Shenzhou Pharmaceutical Co (Zhejiang, China), respectively, while L-thyroxine hormone was supplied by Azico Biophore Ltd (Pradesh, India). Stock solution (100x intestinal concentration) of each drug compound was prepared by dissolving drugs using the least amount of Dimethyl Sulfoxide (DMSO) and diluted with sterile distilled water. Drug stocks were stored at -20°C.

The intestinal concentrations used were previously calculated by Maier and colleagues (2018) and the used final concentrations were 0.562, 211.99, and 0.0481µM for ethinyl estradiol, progesterone and L-thyroxine, respectively (Maier et al., 2018).

### 2.3 Screening the effect of hormonal drugs on bacterial growth

This assay was carried out according to Maier et al. (2018) with some modifications. The bacterial strains were cultured on their specific medium, and then one isolated colony was allowed to grow overnight on modified Gifu anaerobic (mGAM) broth at 37°C under anaerobic conditions and that was repeated twice to ensure that the culture was robust and consistent. The bacterial overnight culture was adjusted to reach starting OD_600_ of 0.02. Two controls were used simultaneously along with the test for this experiment. Control (1) bacteria in medium, control (2) bacteria in medium containing DMSO at a final concentration as in diluted drugs. The effect of DMSO at final concentrations on the growth of bacterial strains was monitored using controls (1) and (2).

The drug stocks were thawed, and an aliquot was diluted to reach 2x intestinal concentrations. In a 96-well flat bottom plate (Greiner Bio-one^®^, Germany), 50 µl of the adjusted bacterial suspension (OD_600 =_ 0.02) were added to 50 µl of the drug concentration (2x). Plates were incubated at 37°C under anaerobic conditions. For the viable count of bacterial growth at zero and 24 hours, tenfold serial dilutions (10^-1^-10^-6^) were performed. An aliquot (10 µl) was spotted on NABA, MRS supplemented with cystine (MRS-C), MacConkey agar, and LBS for the enumeration of *B. fragilis, B. longum, E. coli*, and *L. reuteri*, respectively. The plates were incubated at 37°C under anaerobic conditions for 48 hours. Following incubation, colonies were counted and used to calculate the number of colonies per ml (CFU/ml) (Wang et al., 2017; Maier et al., 2018).

### 2.4 Screening the effect of hormonal drugs on auto-aggregation and cell surface hydrophobicity

The tendency of identical bacterial cells for self-adherence (auto-aggregation) and cell surface hydrophobicity (CSH) are two independent traits that influence bacterial adhesion ability to surfaces (Rahman et al., 2008).

#### 2.4.1 Preparation of bacterial inoculum

*L. reuteri, B. fragilis*, *B. longum*, and *E. coli* were grown in a 5 ml broth of MRS, BHIS, RCM, and LB, respectively, with different drugs at their intestinal concentrations. Tubes were incubated under anaerobic conditions for 18 hours at 37°C. Positive control was run simultaneously where bacteria were cultured in media with a DMSO concentration equivalent to that used in dissolving the drugs. Bacterial pellets were harvested by centrifugation at 9500 rpm for 10 min at 18°C followed by washing twice with ice-cold phosphate buffer saline to be used in the auto-aggregation and cell surface hydrophobicity (Botes et al., 2008).

#### 2.4.2 Auto-aggregation

Bacterial cells were suspended in saline and adjusted to OD_600_ of 0.3. One milliliter of the adjusted bacterial suspension was transferred into a sterile eppendorf tube, then centrifuged at 2000 rpm for 2 min (Botes et al., 2008). The supernatant’s optical density (OD_600_) was measured immediately (A_0_) and after one hour (A_60_)(Botes et al., 2008). Auto-aggregation percentage was calculated using the following equation:


$$\%\;Autoaggregation=\left[\frac{\left(\text{A}0\right)-\left(\text{A}60\right)}{\left(\text{A}0\right)}\right]\text{ x }100\;$$

#### 2.4.3 Cell surface hydrophobicity

The assay of microbial adhesion to hydrocarbons was used to characterize microbial hydrophobicity according to Lather and his colleagues (2016) with some modifications. Bacterial cells were suspended in saline and adjusted to OD_600_ = 0.5. In a glass tube, 0.8 ml volume of xylene was added to 4.8 ml of the adjusted bacterial suspension. The mixture was shaken vigorously for 1 min and allowed to separate at room temperature for 60 min. Bacterial cells were distributed between aqueous and organic phases according to bacterial hydrophobicity. The aqueous phase was removed with caution using a micropipette and measured by spectrophotometer at wavelength 500 nm (A) (Lather et al., 2016). Percentage hydrophobicity was calculated using the following equation:


$$\%\;Hydrophobicity=\left(1-\frac{A}{A0}\right)x\;100$$

A0 is the absorbance before the addition of xylene while A is the absorbance in the aqueous phase after the addition of xylene.

### 2.5 Screening the drug activity on biofilm formation

The effect of drugs on biofilm formation was measured by crystal violet assay. A selective medium supplemented with 1% glucose was used for the incubation of each bacterium at 37°C for 24 hours under anaerobic conditions. Peptone yeast glucose (PYG), LB, BHIS, and RCM were used for the assessment of *L. reuteri*, *E. coli*, *B. fragilis*, and *B. longum*, respectively. The cell density of bacterial suspension was adjusted to OD600 = 1, followed by dilution 1:100 using the selective fresh medium for each bacterium. In a 96-well flat bottom plate (Greiner Bio-one^®^, Germany), 100 µl of the bacterial cell suspensions were inoculated with 100 µl of drugs (2x) in each well, incubated for 48 hours at 37°C under anaerobic conditions. After incubation, the bacterial growth was measured using a microtiter plate reader (STAT FAX 2200, Awareness Technology, Florida, USA) at a wavelength of 630 nm. Adhered cells were washed and then stained with 0.1% crystal violet for 30 min followed by washing and solubilization. The colored solution was measured at wavelength 545 nm. The readings were used to calculate the biofilm formation index (Kwasny and Opperman, 2010; Coffey and Anderson, 2014; Woo et al., 2017; Jang et al., 2020). Five technical replicates were used for each bacterial strain to compensate for variability and three biological replicates were performed. A positive control with bacteria in addition to DMSO was used simultaneously along with the test.


$$Biofilm\;formation\;index=\frac{\left(OD\;545-OD\;control\right)}{\left(OD\;630-OD\;control\right)}$$

OD 545 is colorimetric absorbance of stained bacteria, OD 630 is absorbance of bacterial growth, and OD control is absorbance of negative control.

### 2.6 Screening the effect of selected drugs on bacterial adherence to cell lines

Co-culture cells of Caco-2/HT29 (90:10) was used to simulate intestinal tissue which was prepared according to the method described by Kleiveland (2015).

#### 2.6.1 Cytotoxicity assay of tested drug on cell line

The effect of drugs and DMSO on cell line viability was measured using 3, -4,5 dimethyithiazol-2,5 diphenyl tetrazolium bromide (MTT) assay. Co-culture cells of Caco-2/HT29 were seeded in 96 well microtiter flat bottom plate (Greiner Bio-one^®^, Germany) using Roswell Park Memorial Institute (RPMI) medium supplemented with 2% Fetal bovine serum (FBS) and incubated overnight in 5% CO_2_ at 37°C. Following incubation, 100 µl of each drug in their working dilution was added to co-culture cells (three technical replicates for each concentration). A control was run simultaneously: a negative control with DMSO concentrations equivalent to that in drug solution. The plates were sealed and incubated overnight in 10% CO_2_ at 37°C. MTT was dissolved in fresh medium at concentration 0.05%, added to each well, and incubated for 2 hours under the same conditions. After incubation, the medium was aspirated, and 100 µl of DMSO was used for solubilization. Color was measured at wavelength 545 nm and readings were used to calculate percentage viability (Mueller et al., 2004).


$$\%\;Cell\;Viability=\frac{Average\;OD\;of\;Drugs\;or\;DMSO\;treated\;cell\;lines\;coculture}{Average\;OD\;of\;untreated\;cell\;lines\;coculture\;}\;\times 100$$

#### 2.6.2 Adherence to cell line assay

The bacterial strains were cultured on their specific media for 20 hours under anaerobic conditions at 37°C. Bacterial cells were centrifuged at 6000 rpm for 5 min at 4°C, the pellet was washed twice with PBS and the cell density for each bacterium was adjusted to 1x10^8^ CFU/ml using PBS. In a 24 well flat bottom plate (Greiner Bio-one^®^, Germany), Caco-2/HT-29 co-culture was grown and maintained using RPMI medium except for *E. coli* where DMEM medium was used. On plates seeded with cell line coculture, 100 µl of adjusted bacterial suspension and 100 µl of drugs (2x intestinal concentration) were added. Thus, final concentration of the drugs in each well will be equivalent to intestinal concentration (1x). Plates were sealed and incubated at 37°C for 2 hours in 5% CO_2_. Following incubation, the medium was removed, and wells were washed using 200 µl of fresh medium to remove non-adherent cells. The cells were then lysed by adding 100 µl of 0.1% Triton X-100 for 10 min at room temperature, then the reaction was stopped by addition of 900 µl of fresh medium. Viable bacterial cells were enumerated by spreading 10µl of diluted bacterial culture (drop plate technique) on their selective medium prior to incubation with cell line (CFU of initial inoculum) and after incubation for 2 hours (CFU of adhered cells)(Letourneau et al., 2011; Gagnon et al., 2013; Reddy and Austin, 2017). The count was recorded and used to calculate the percentage of adhered bacteria.


$$\%\;adhered\;cells=\frac{CFU\;of\;adhered\;cells\;\left(2\;hours\right)}{CFU\;of\;initial\;incoulum\;\left(0\;hour\right)}\;\times 100$$

#### 2.6.3 Imaging of cell adherence to CaCo-2/HT29 co-culture using scanning electron microscope

The same steps of adherence assay were followed for preparation of samples in a 12 well flat bottom plate (Greiner Bio-one^®^, Germany) for imaging the cell adherence using scanning electron microscope. After incubation for 2 hours, the plate was washed with phosphate buffer saline (PBS) twice, and 5% glutaraldehyde (prepared in 0.1M sodium cacodylate) was added for 2 hours for fixation. The wells were dehydrated by passing them through graded ethanol (25, 50, 70, 80, and 90%) for 10 min in each concentration at room temperature. The last concentration used for dehydration was 100% for 15 min. The wells were coated with gold and examined using scanning electron microscope (Quanta 250 FEG, West Bengal, India) with a magnification power of 5000x and 10000x (Heckman et al., 2007; Ude et al., 2019).

### 2.7 Measurement of the change in Short Chain Fatty Acids and lactic acid production by bacteria under the effect of hormonal drugs using high performance liquid chromatography (HPLC)

Strict anaerobic bacteria are known for their ability to produce short-chain fatty acids by the saccharolytic fermentation of complex polysaccharides (Nogal et al., 2021), therefore the effect of hormonal drugs on production of SCFA by *B. fragilis* and *B. longum* were studied. Analytical samples were prepared by inoculating colonies of *B. fragilis* and *B. longum* in BHIS broth and MRS broth, respectively for 48 hours at 37°C under anaerobic conditions. The bacterial OD was adjusted to 0.01 at 600 nm and incubated with the intestinal concentration of the three hormonal drugs at 37°C for 16 hours under anaerobic conditions. The suspension was then centrifuged at 9500 rpm for 15 min at 4°C and the supernatant was collected. The analytical sample was injected into the HPLC system (Smart line, Knauer, Germany) using the autosampler after its conditions was set. The HPLC system was equipped with Rezex™ column (Phenomenex, California, USA) for organic acid analysis. The flow rate was set at 0.6 ml/min, UV detector set at 214 nm, column oven temperature kept constant at 65°C, and the mobile phase was 0.005M H_2_SO_4_. To create the standard curve, a standard solution containing lactic, acetic, propionic, and butyric acids was prepared at concentrations of 1, 10, 100, 500, and 1000 ppm. The SCFA quantities were determined using the standard curves’ appropriate linear regression equations (R^2^ ≥ 0.99). The response factor is a measurement of the analyte’s relative spectral response to its external standard at the specified retention time, followed by calculation of SCFAs in ppm. Positive control was prepared by growing bacteria in media containing DMSO in concentrations equivalent to their final concentration in drug solution. The data was integrated by clarity chrom software (DataApex, Praha, Czechia)

### 2.8 Statistical analysis

GraphPad Prism 9.1.1 (GraphPad Software Inc., CA, USA) was used for statistical analysis. Multiple unpaired t-tests and multiple comparisons using the Holm-Šídák method were used to compare auto-aggregation, hydrophobicity, and formation of biofilm in the presence and absence of drugs. For statistical analysis of the viable count of the microorganisms in the screening of the antibacterial activity of drugs and adherence assay to cell lines coculture, the Mann-Whitney t-test was used. An unpaired t-test was used for the statistical analysis of the effect of drug on the viability of the intestinal cell lines. The readings were considered significant at p<0.05.

## 3 Results

### 3.1 Alteration in bacterial growth under the effect of hormonal drugs

**Figure 1:**
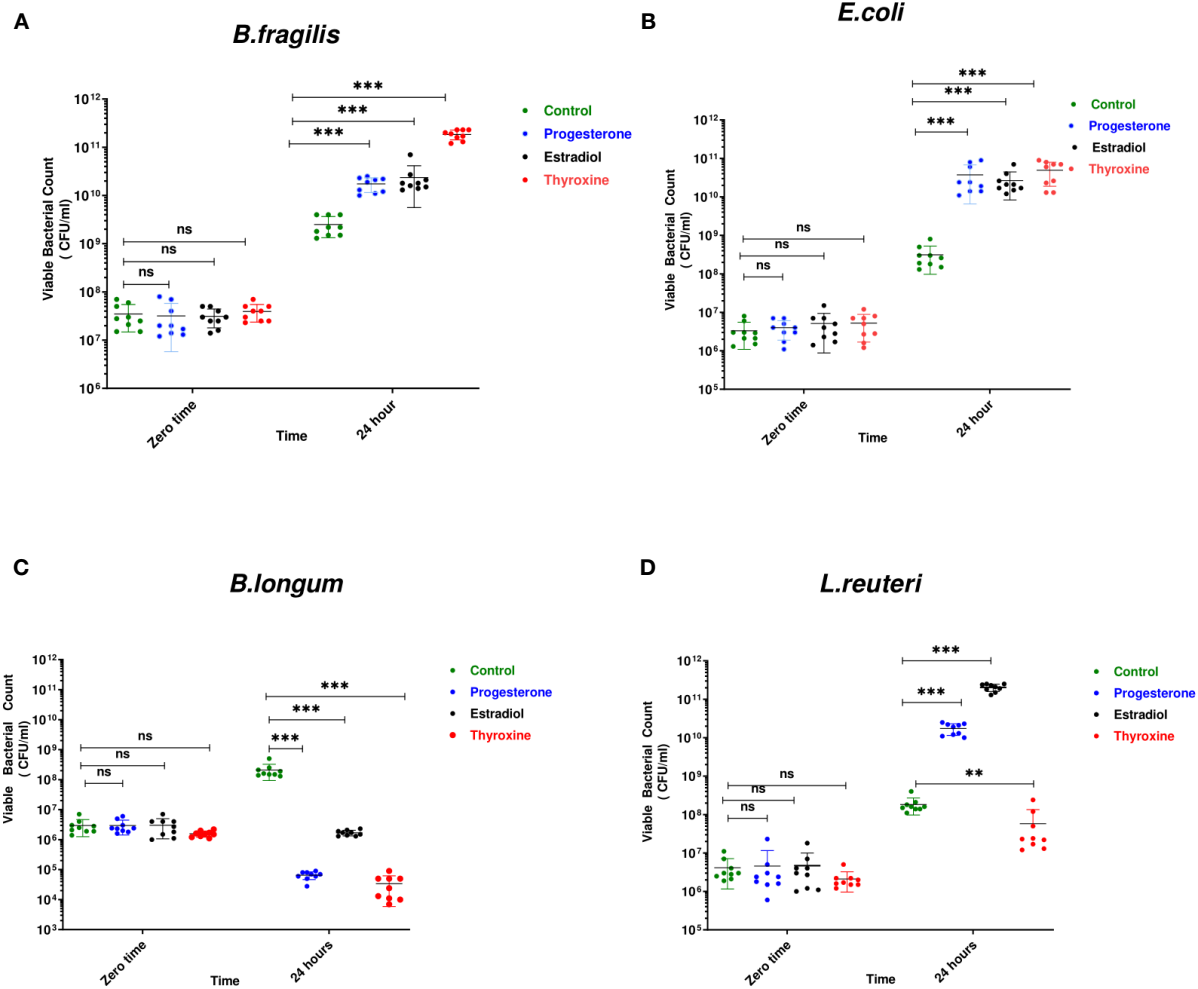
Growth of bacteria under the effect of hormonal drugs. Effect of hormonal drugs on the growth of: Gram-negative bacteria **(A)** *Bacteroid fragilis*, **(B)** *Escherichia coli*, and Gram-positive bacteria **(C)** *Bifidobacterium longum*, **(D)** *Limosilactobacillus reuteri* represented as viable count (CFU/ml). Tested hormonal drugs include progesterone, ethinyl estradiol, and L-thyroxine in their intestinal concentrations, 211.99, 0.562, and 0.0481µM, respectively. Control represents the growth of bacteria in addition to DMSO. Mann-Whitney t-test was used to statistically compare the effect on bacterial growth. Significance level of **(P<0.001), ***(P<0.0001). ns, non significant.

*B. longum* was the most affected bacteria in presence of the three tested hormonal drugs showing reduction in viable count by 3, 2, and 4 logs in presence of progesterone, estradiol, thyroxine, respectively **(** **Figure 1C** **)**. The growth of Gram-negative bacteria was enhanced by hormonal treatment showing increase by 1 to 2 logs by *B. fragilis* **(** **Figure 1A** **)** and *E. coli* **(** **Figure 1B** **)**. The effect on the growth of *L. reuteri* was variable where the steroid hormones increased its growth by 2 to 3 logs while thyroxine reduced its growth by one log **(** **Figure 1D** **)**.

### 3.2 Progesterone changed the auto-aggregation of tested bacteria

**Figure 2:**
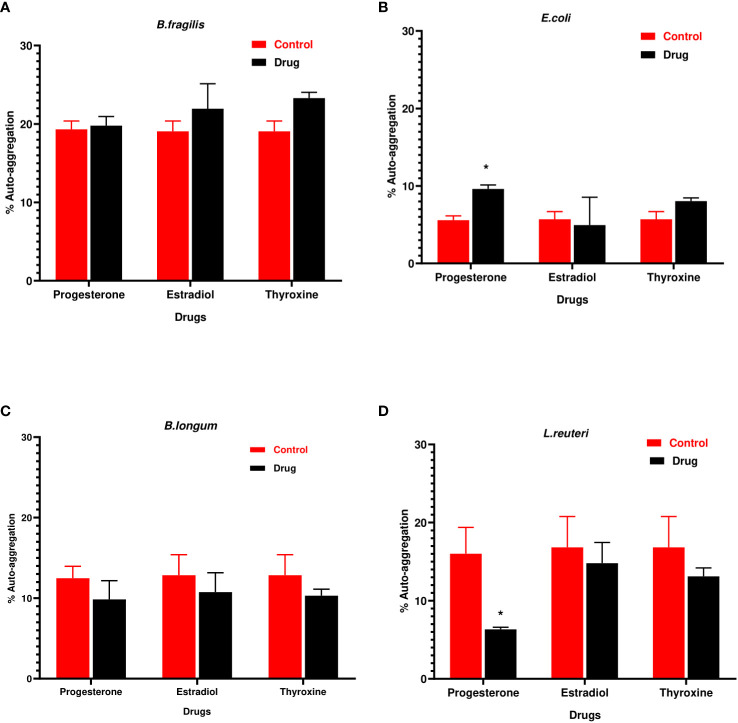
Auto-aggregation of bacteria with hormonal drugs. Change in percentage auto- aggregation of **(A)** *B. fragilis*; **(B)** *E. coli;* **(C)** *B. longum;* **(D)** *L. reuteri* after incubation with hormonal drugs at 37°C for 60 min. Values were expressed as the mean of the percentage of three experiments with error bars (SE). Tested hormonal drugs include progesterone, ethinyl estradiol, and L-thyroxine in their intestinal concentrations, 211.99, 0.562, and 0.0481µM, respectively. Control represents the bacteria in addition to DMSO. Multiple unpaired t-tests along with Holm-Šídák for multiple corrections were used to statistically compare the effect of different drugs on bacterial auto-aggregation. * Significant difference (p<0.05).

Progesterone was the only hormonal drug under test that showed an effect on bacterial auto-aggregation **(** **Figures 2A–D** **)** as it caused a significant increase (P=0.0008, α=0.05) in the auto-aggregation of *E.coli* from 5.5% in control to 10% in presence of drug **(** **Figure 2C** **).** On the other hand, progesterone remarkably reduced (P=0.007, α=0.05) the auto-aggregation of *L. reuteri* cells to 5% compared to the control **(** **Figure 2D** **)**.

### 3.3 Hormonal drugs change cell surface hydrophobicity of tested bacteria

**Figure 3:**
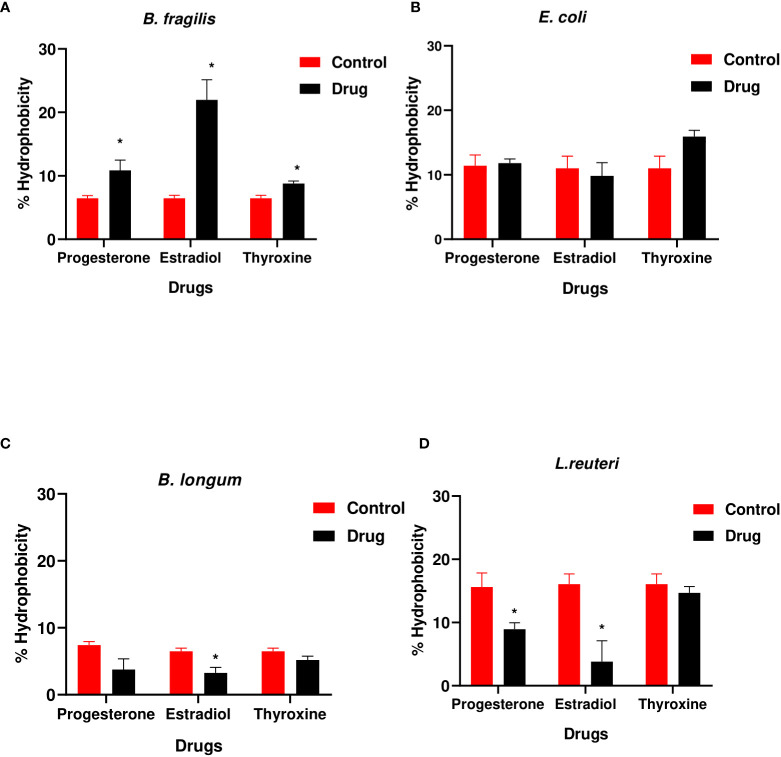
Hydrophobicity of bacteria under the effect of hormonal drugs. Change in percentage hydrophobicity of **(A)** *B. fragilis*; **(B)** *E. coli;* **(C)** *B. longum;* **(D)** *L. reuteri* with hormonal drugs after incubation at 37 °C for 60 min. Values were expressed as the mean of the percentage of three experiments with error bars (SE). Tested hormonal drugs include progesterone, ethinyl estradiol, and L-thyroxine in their intestinal concentrations, 211.99, 0.562, and 0.0481µM, respectively. Control represents the bacteria in addition to DMSO. Multiple unpaired t-test along with Holm-Šídák for multiple corrections was used to statistically compare the effect of different drugs on bacterial hydrophobicity. * Significant difference (p<0.05).

The hydrophobicity of *B. fragilis* increased significantly (P<0.01) in the presence of the three hormonal drugs (**Figure 3A**). Estradiol reduced significantly (P<0.01) the hydrophobicity of *B. longum* and *L. reuteri* while progesterone reduction to hydrophobicity was limited to *L. reuteri* (**Figures 3C, D**). *E. coli* did not show significant change in hydrophobicity in presence of the three drugs **(** **Figure 3B** **).**

### 3.4 Hormonal drugs changed biofilm formation ability of tested bacteria

**Figure 4:**
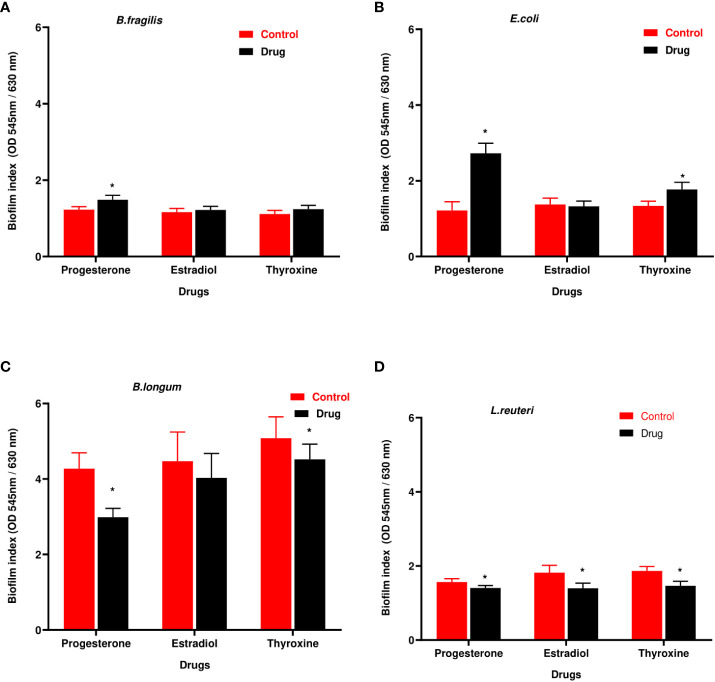
Biofilm formation ability of selected bacteria under the effect of hormonal drugs. Change in biofilm formation index by **(A)** *B. fragilis*; **(B)** *E. coli* **(C)**; *B. longum;* **(D)** *L. reuteri* with hormonal drugs. Values were expressed as the mean of the percentage of three experiments with error bars (SE). Tested hormonal drugs include progesterone, ethinyl estradiol, and L-thyroxine in their intestinal concentrations, 211.99, 0.562, and 0.0481µM, respectively. Control represents the bacteria grown in culture with DMSO. Multiple unpaired t-test along with Holm-Šídák for multiple corrections was used to statistically compare the effect of different drugs on bacterial biofilm. * Significant difference (p<0.05).

*B. longum* showed the highest biofilm index among tested isolates. Hormonal drugs increased biofilm formation by Gram-negative bacteria (**Figures 4A, B**) but reduced the ability of Gram-positive bacteria to form biofilm (**Figures 4C, D**).

### 3.5 Effect of tested drugs on bacterial adherence to Caco-2/HT-29 coculture cell lines

An unpaired t-test was used to compare the difference in cell viability in presence of drugs compared with the untreated coculture cell lines and no significant effect was observed on coculture viability when treated with the three drugs in their intestinal concentrations (data not shown).

**Figure 5:**
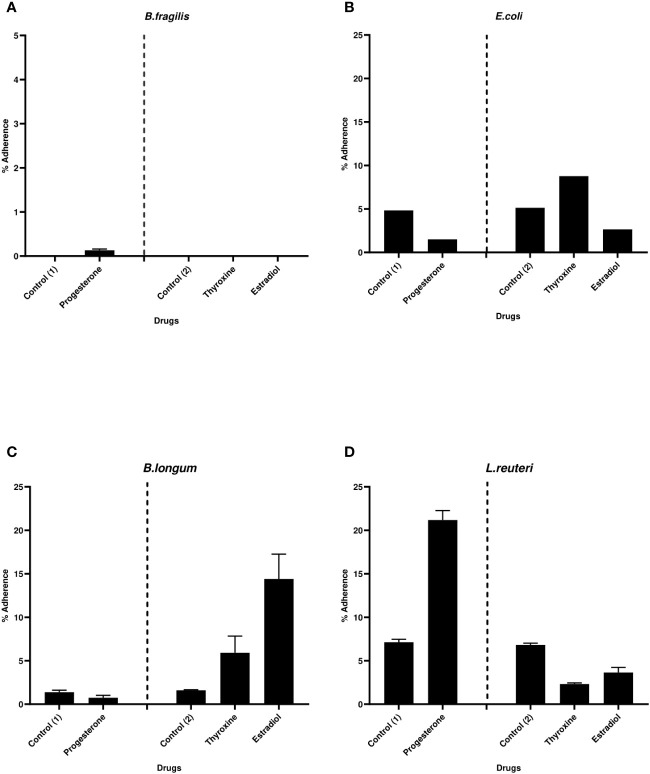
Bacterial adherence to Caco-2/HT-29 co-culture under the effect of hormonal drugs. Change in the percentage of adhered bacterial cells **(A)** *B*. *fragilis*; **(B)** *E. coli*; **(C)** *B*. *longum*; **(D)** *L. reuteri* under the effect of hormonal drugs: progesterone, ethinyl estradiol, and thyroxine in their intestinal concentrations 211.99, 0.562, and 0.0481µM, respectively. Values were expressed as the mean of the percentage of three experiments with error bars (SE). Positive control with bacteria and DMSO at different concentrations equivalent to that used to dissolve drug ( control (1): DMSO 1% and Control (2):DMSO 0.001%).

The effect of hormonal drugs on adherence of tested bacteria was variable. No growth of *B. fragilis* was observed on NABA after 2 hours of incubation in both control and drug-treated samples except with progesterone which showed relatively low adherence of 0.13% **(** **Figure 5A** **).** Progesterone reduced the adherence of *E. coli* and *B. longum* while increasing the adherence of *L. reuteri* (**Figures 5B–D** **)**. Thyroxine remarkably increased the percentage of adhered *E. coli* and *B. longum* to cell lines coculture while reducing adherence of *L. reuteri* when compared to control. Ethinyl estradiol reduced the adherence of *E. coli* and increased the adherence of *B. longum* and *L. reuteri*.

### 3.6 Scanning electron microscope

**Figure 6:**
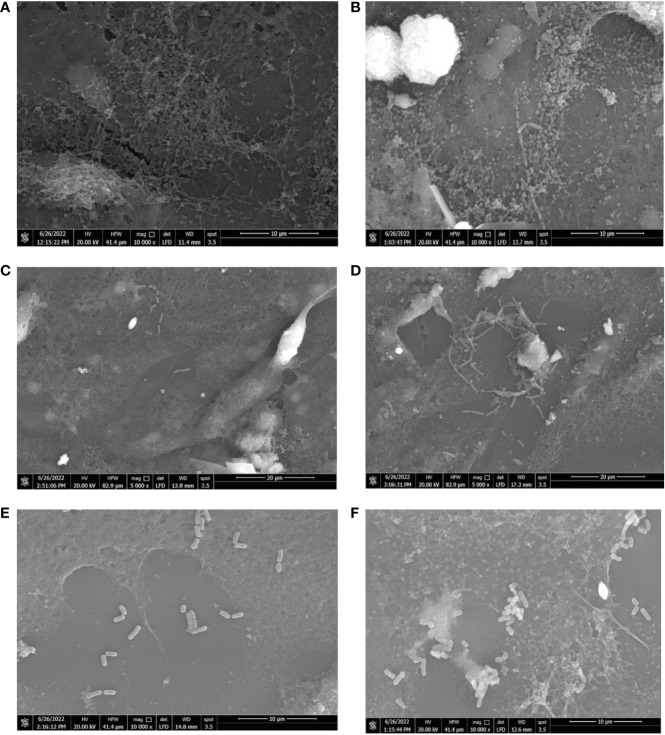
Scanning electron micrograph showing the effect of hormonal drugs on adherence of bacteria to Caco-2/HT-29 co-culture. Effect of progesterone on *B*. *fragilis* adherence **(A)** Control untreated bacterial cells **(B)** Drug-treated bacterial cells (Magnification power 10000x); *B longum* **(C)** Control untreated bacterial cells **(D)** Drug-treated bacterial cells (Magnification power 5000x); *L. reuteri* **(E)** Control with bacterial cells **(F)** Drug-treated cells bacterial cells (Magnification power 10000x). The adherence assay was carried out using RPMI medium in 5% CO_2_ at 37°C, and imaging was performed using a scanning electron microscope (Quanta 250 FEG, West Bengal, India).

The influence of hormonal drugs on bacterial adherence was confirmed by SEM images. As shown in **Figure 6A**, untreated *B. fragilis* showed no visible bacterial attachment to the extracellular matrix, while exposure to progesterone increased adherence to the cell line which appeared as few longitudinal rods attaching to the cell line co-culture in **Figure 6B**. Ethinyl estradiol increased the number of *B. longum* bacteria adhering to cell line coculture **(** **Figure 6D** **)** when compared to the control **(** **Figure 6C** **)**. The effect of progesterone on the adherence of *L. reuteri* in the presence **(** **Figure 6F** **)** and absence of the drug **(** **Figure 6E** **)** where a visible slight increase in the number of *L. reuteri* was observed in treated cells.

### 3.7 Alteration in SCFAs and lactic acid production by *B. fragilis* and *B. longum* in presence of hormonal drugs

The reference chromatograms obtained from the standard solution revealed that SCFAs and lactic acid were detected at different retention times: 13.100, 14.876, 17.967, and 20 min; for lactic, acetic, propionic and butyric acids, respectively. The amount of butyric acid produced wasn’t detected in treated and untreated samples under the test conditions. The response factor was calculated for each SCFA, which represented the measurement of the analyte’s relative spectral response to its external standard. The highest acid produced by *B. fragilis* was acetic acid followed by lactic acid then propionic acid. The interpretation of chromatogram (**Supplementary Figure 1** and **Supplementary Table 1**) showed a reduction in the amount of both lactic acid and propionic acid and an increase in the amount of acetic acid produced by *B. fragilis* in presence of progesterone (**Table 1** **)**. The concentration of lactic acid and acetic acid produced by *B. fragilis* was reduced after being treated with ethinyl estradiol, conversely an increase in the amount of propionic acid was observed under the effect of the same drug. Both lactic acid and propionic acid produced by *B. fragilis* increased in amount when the bacteria were treated with thyroxine hormone while acetic acid levels were reduced under the same conditions.

**Table 1:** The concentration of SCFAs and lactic acid produced by *Bacteroides fragilis* under the effect of hormonal drugs (progesterone, ethinyl estradiol, and thyroxine) measured by HPLC.

Drugs	Compound	Concentration (ppm)	
		Control*	Sample
Progesterone	Lactic acid	1738.136	411.882 ↓
	Acetic acid	5661.760	5809.819 ↑
	Propionic acid	409.485	253.075 ↓
Ethinyl estradiol	Lactic acid	986.53	376.539 ↓
	Acetic acid	5138.918	4933.959 ↓
	Propionic acid	347.249	497.194 ↑
Thyroxine	Lactic acid	986.53	1218.416 ↑
	Acetic acid	5138.918	4512.039 ↑
	Propionic acid	347.249	403.225 ↓

* Positive control of bacteria in media containing DMSO in concentrations equivalent to their final concentration in drug solution.

↑increase production compared to control.

↓lower production compared to control.

Propionic and butyric acid weren’t detected in *B. longum* control or drug-treated samples. The chromatogram (**Supplementary Figure 2** and **Supplementary Table 2**) showed a reduction in the amount of both lactic acid and acetic acid produced by *B. longum* when treated with progesterone. *Bifidobacterium longum* produced higher levels of both lactic acid and acetic acid after being treated with ethinyl estradiol. Both lactic acid and butyric acid produced by *B. longum* increased in amount when the bacteria were treated with thyroxine hormone **(** **Table 2** **).**

**Table 2:** The concentration of SCFAs and lactic acid produced by *Bifidobacterium longum* under the effect of hormonal drugs (progesterone, ethinyl estradiol, and thyroxine) measured by HPLC.

Drugs	Compound	Concentration (ppm)	
		Control*	Sample
Progesterone	Lactic acid	315.68	116.640 ↓
	Acetic acid	7994.02	7296.440 ↓
Ethinyl estradiol	Lactic acid	289.266	331.122 ↑
	Acetic acid	8133.807	8405.94 ↑
Thyroxine	Lactic acid	986.53	309.830 ↓
	Acetic acid	5138.918	8322.263 ↑

* Positive control of bacteria in media containing DMSO in concentrations equivalent to their final concentration in drug solution.

↑increase production compared to control.

↓lower production compared to control.

## 4 Discussion

The human gastrointestinal (GI) tract is a niche to a complex and dynamic community of bacteria known as the gut microbiota, which has a significant impact on the host during health and disease (Thursby and Juge, 2017). The composition and function of the gut microbiome are also influenced by different factors including the use of medications (Wen and Duffy, 2017).

In our study, the steroid hormones such as ethinyl estradiol and progesterone significantly increased the growth of *B. fragilis, E. coli*, and *L. reuteri* whereas decreasing the growth of *B. longum.* Conversely, previous studies reported the antibacterial effect of steroid derivatives by preventing the normal development of the cell membrane integrity and permeability. Thus, it is thought that the reason for the antibacterial effect of steroid bile acids is due to their binding to phospholipids in bacterial membranes resulting in membrane destruction and ultimately cell death (DoĞAn et al., 2017; Bustos et al., 2018; Vollaro et al., 2020; Crowley et al., 2022). The resistance of the selected gut bacteria to the deleterious effect of steroids could be due to the presence of bile salt hydrolase enzyme in these genera which protect them from the damaging effect of steroid bile acids (Staley et al., 2017). Another explanation for the resistance of *L. reuteri* to bile salts was the protective effect of this bacteria against steroids as bile acids arising from precipitation of the deconjugated bile salts and physical binding of bile salts by a bacterium, so rendering the detrimental bile salts accessible (De Boever et al., 2000). The increase in growth of *B. fragilis* could be explained by the study carried out by Kornman and Loesche (1982) using labeled C^14^ steroid hormones who proved the uptake of these hormones by *Bacteroides* bacteria and explained their ability to substitute vitamin K compounds, an essential growth factor, with progesterone and ethinyl estradiol resulting in an increased growth curve (Kornman and Loesche, 1982). Both steroid hormones had a significant effect in reducing the growth of *B. longum* in this study supported by findings of previous studies on Gram-positive bacteria, which demonstrated that different steroids reduce *in vitro* growth and increase cell leakage (Souza et al., 2021). *B. longum* growth was reduced under the same treatment which could be attributed to their lower resistance to bile acids compared to other *Bifidobacterium* species (Ibrahim and Bezkorovainy, 1993; Clark and Martin, 1994; Dunne et al., 2001). Many cross sectional studies for the influence of sex steroids on gut bacteria have proved the existence of correlation between sex hormone levels and microbiota composition despite the inevitable interfering factors, including genetics and environment (Santos-Marcos et al., 2018; Shin et al., 2019; Zhao et al., 2019; He et al., 2021). Researchers reported that lower levels of estradiol in postmenopausal women and men is accompanied by increase in *Bacteroidetes* sp. and depletion of *Lactobacillus* sp. Compared to women with higher levels of sex hormones (Santos-Marcos et al., 2018; Zhao et al., 2019). Likewise, the direct effect of the estradiol hormone on the growth of *L. reuteri* has been proved in our study. Treatment of sex steroid deficient mice with *Lactobacillus rhamnosus* could avoid bone loss which indicates the importance of this bacterium in preserving bone density (Li et al., 2016). The effect of sex steroids on gut bacteria *in vivo* could not be compared by their effect *in vitro* because, in some cases, hormonally related microbial shifts result from endogenous steroid-induced tissue and immunological changes rather than from steroids’ direct effect on bacteria (Lester and Hechter, 1958; Feraco et al., 2016).

While studying the effect of L-thyroxine on the viability of gut microbiota, results revealed a significant increase in the growth of Gram-negative *B. fragilis* and *E. coli* and a significant reduction in the growth of Gram-positive *B. longum* and *L. reuteri*. The results withstood the previous findings by Garber and Lupowitz-Donenfeld (1973) that L-thyroxine had an inhibitory effect on the growth of Gram-positive bacteria however, it has no significant effect on Gram-negative bacteria. The inhibition of Gram-positive bacteria by L-thyroxine was reduced by cations such as Mn^2+^, Fe^2+^, and Ca^2+^ which support the hypothesis that L-thyroxine chelating effect is one of the factors contributing to its inhibitory effect (Garber and Lupowitz-Donenfeld, 1973; Benvenga et al., 2017). Metagenomic analysis of gut microbiome in hyperthyroidism patients showed as a significant decline in *Bifidobacterium* and *Lactobacillus* (Zhou et al., 2014). Similarly, reduction in these two genera was detected in our study under the influence of L-thyroxine which also explain the need for probiotic supplement in hypothyroidism patients treated with L-thyroxine to keep bone density and optimizing thyroid function (Knezevic et al., 2020).

The process of bacterial adhesion to different surfaces is a complex process that involves contact between bacterial membranes and interacting surfaces. Specific and nonspecific binding are the two essentially distinct strategies that cause bacterial adhesion (Piette and Idziak, 1992). Electrostatic or hydrophobic interactions have a major role in the non-specific binding and considerably affect adhesion strength (Piette and Idziak, 1992). Two factors were assessed in this study to understand the influence of their changes in altering bacterial adherence in the presence of hormones. The auto-aggregation and cell surface hydrophobicity assays were carried out to evaluate the effect of the drugs on non-specific binding of these bacteria to different surfaces. Previous study showed that biofilm formation is correlated with hydrophobicity (Krasowska and Sigler, 2014) however this correlation was detected in *L. reuteri* under the effect of tested drugs where reduction in hydrophobicity was accompanied by the reduction in biofilm formation ability of this bacterium. Conversely, hydrophobicity was not correlated to bacteria adherence to Caco-2/HT-29 co-culture. Similarly, some previous studies showed that the correlation between hydrophobicity and adhesion to hydrophobic mucosal cells was strain specific (Kos et al., 2003; Muñoz-Provencio et al., 2009). This has suggested that nonspecific binding factors such as hydrophobicity is not an accurate measure of adhesive potential to enterocytes (Van Tassell and Miller, 2011) and interactions between microbes and hosts depend greatly on the structure of the cell surface rather than nonspecific binding (Nishiyama et al., 2021). Many studies have shown that, components of a protein nature such as mucus adhesins in *L. reuteri* (Vélez et al., 2007; Sánchez et al., 2008) and *B. longum* (Izquierdo et al., 2008) as well as capsular polymer in *B. fragilis* (Nakano et al., 2008; Reis et al., 2014) are primarily important for bacterial adherence to intestinal mucin types and/or epithelial cells beside saccharide moieties and lipoteichoic acid.

A previous study linked hormonal drugs’ impact on bacterial growth with its impact on bacterial biofilm (Fteita et al., 2014). Similarly, in our results, a change in bacterial growth under the effect of the drug was associated with a similar change in biofilm formation except *L. reuteri* showing a reduction in biofilm in presence of sex steroids despite the increase in growth and this could be explained by the increased production of biosurfactant by *Lactobacillus* species in the presence of sex steroids thus decreasing biofilm formation (Clabaut et al., 2021). Additionally, the effect of progesterone on quorum sensing has been reported for some bacteria which could affect phenotypes that depend on cell-to-cell communication (Cadavid et al., 2018).

The gut microbiota makes use of SCFAs as a source of carbon by cross-feeding, but SCFAs can be harmful to some gut bacterial species when present in high concentrations (Feng et al., 2018). The amount of SCFAs produced by the anaerobic bacteria *B. fragilis* and *B. longum* was measured using HPLC. Surprisingly we did not detect butyric acid after incubation of *B. longum* for 48 hours despite being known as butyric acid producer (Aguirre et al., 2016). Additionally, propionate was not detected in *B. longum* culture and similar result was obtained by Rios-Covian et al. (2017). The effect of the tested drug compounds in this study on SCFAs production was variable and mostly independent of their effect on bacterial growth, in contrast to earlier studies’ results that a change in abundance of gut bacteria owing to drug usage was correlated to a similar effect on SCFAs production (Hojo et al., 2018). Notably, numerous studies reveal that *Bacteroidetes* are the main producers of propionate in the human gut (Salonen et al., 2014; Aguirre et al., 2016) and this SCFA is known for its ability to suppress inflammation and the increase in its level is associated with cognitive decline in elderly (Kawasoe et al., 2022; Neuffer et al., 2022) while low level of propionic acid and acetate was linked to autism(Tetel et al., 2018). Progesterone and L-thryoxine reduced the production of propionic acid by *B. fragilis*. A key organic acid in the fermentation of prebiotics is lactic acid, which is generated in the GIT by the bacteria *Lactobacilli* and *Bifidobacteria*. Lactic acid does not significantly accumulate in the intestinal lumen, and it is further metabolized by cross-feeding species, particularly with the butyrate-producing bacteria, to acetate or butyrate, or propionate.

## 5 Conclusion

Oral hormonal drugs can affect the growth, biofilm formation and adherence of gut bacteria at intestinal concentrations *in vitro* which can explain specific microbiome signatures associated with long term use of these drugs. Steroid hormones increase the growth of *B. fragilis*, *E. coli*, and *L. reuteri* while reducing the growth *B. longum.* However, thyroxine increased the growth of Gram-negative bacteria and reduced the growth of Gram-positive one. The effect of these drugs on biofilm formation by selected bacteria was linked to their effect on growth except *L. reuteri* where steroid hormones reduced biofilm formation by this bacterium despite increasing bacterial growth. The effect of hormonal drugs on bacterial adherence to Caco-2/HT-28 coculture was not solely dependent on the change in hydrophobicity but other specific binding factors might contribute to their effect. The effect of the tested drug compounds on SCFAs and lactic acid production was variable and mostly independent of their effect on bacterial growth.

## Data availability statement

The original contributions presented in the study are included in the article/**Supplementary Material**. Further inquiries can be directed to the corresponding author.

## Author contributions

NA and RW conceptualized the work. ZH performed the experiments.NA, RW, and ZH analyzed the results. All authors contributed to the article and approved the submitted version.
