# Supplementary material for: Hormonal drugs: Influence on growth, biofilm formation, and adherence of selected gut microbiota
Source: Front Cell Infect Microbiol. 2023 Mar 13;13:1147585. doi: 10.3389/fcimb.2023.1147585 (PMC10042233; doi:10.3389/fcimb.2023.1147585)
Supplement: Supplementary file 1 [file DataSheet_1.docx]

Hormonal drugs: Influence on growth, biofilm formation, and adherence of selected gut microbiota

**Zainab K. Hamouda^1^, Reham Wasfi^1*^, Nourtan F. AbdelTawab^2^**

^1^Department of Microbiology and Immunology, Faculty of Pharmacy, October University for Modern Sciences and Arts, Giza, Egypt

^2^Department of Microbiology and Immunology, Faculty of Pharmacy, Cairo University, Cairo, *** * Correspondence:**Corresponding Author: Dr. Reham Wasfi
rwasfi@msa.edu.eg

# Supplementary Figures and Tables

## Supplementary Figures

|  | Control | Treated |
| --- | --- | --- |
| A |  |  |
| B |  |  |
| C |  |  |
| Supplementary Figure 1*:* HPLC chromatograms showing the effect of hormonal drugs on the production of SCFAs by *Bacteroides fragilis* using Rezex HPLC column. The effect of (A) progesterone, (B) Ethinyl estradiol, and (C) L-thyroxine compared to control (Bacteria were grown with DMSO in concentrations equivalent to the concentrations used to dissolve drugs. The y-axis represents the intensity of absorbance in Milli absorbance unit (mAU) while the x-axis represents the retention time of lactic, acetic, propionic, and lactic acid is 13.100, 14.867, 17.967, and 20.00 min respectively. | | |

|  | Control | Treated |
| --- | --- | --- |
| A |  |  |
| B |  |  |
| C |  |  |
| Supplementary Figure 2*:* HPLC chromatograms showing the effect of hormonal drugs on the production of SCFAs by *Bifidobacterium longum* using Rezex HPLC column. The effect of (A) progesterone, (B) Ethinyl estradiol, and (C) L-thyroxine compared to control (Bacteria were grown with DMSO in concentrations equivalent to the concentrations used to dissolve drugs. The y-axis represents the intensity of absorbance in Milli absorbance unit (mAU) while the x-axis represents the retention time of lactic, acetic, propionic, and butyric acid is 13.100, 14.867, 17.967, and 20.00 min respectively. | | |

## Supplementary Tables

Table 1. The response factor of SCFAs and lactic acid produced by *Bacteroides fragilis* under the effect of hormonal drugs measured by HPLC.

| Drugs | Compound | Response factor * | |
| --- | --- | --- | --- |
|  |  | Control | Sample |
| Progesterone | Lactic acid | 1041.734 | 246.857 ↓ |
|  | Acetic acid | 6791.122 | 6968.715 ↑ |
|  | Propionic acid | 327.588 | 202.460 ↓ |
| Ethinyl estradiol | Lactic acid | 591.271 | 225.678 ↓ |
|  | Acetic acid | 6163.988 | 5918.145 ↓ |
|  | Propionic acid | 277.799 | 397.756 ↑ |
| Thyroxine | Lactic acid | 591.271 | 730.245 ↑ |
|  | Acetic acid | 6163.988 | 5412.064 ↓ |
|  | Propionic acid | 277.799 | 322.580 ↑ |

***** The response factor is the ratio between a signal produced by an analyte and the quantity of analyte which produces the signal.

**↑increase production compared to the control.**

**↓lower production compared to the control.**

Table 2. The response factor of SCFAs and lactic acid produced by *Bifidobacterium longum* under the effect of hormonal drugs measured by HPLC.

| Drugs | Compound | Response factor * | |
| --- | --- | --- | --- |
|  |  | Control | Sample |
| Progesterone | Lactic acid | 280.576 | 69.907↓ |
|  | Acetic acid | 9802.34 | 8751.875 ↓ |
| Ethinyl estradiol | Lactic acid | 173.368 | 198.455 ↑ |
|  | Acetic acid | 9756.274 | 10082.691↑ |
| Thyroxine | Lactic acid | 591.271 | 185.694 ↓ |
|  | Acetic acid | 6163.988 | 9982.321 ↑ |

***** The response factor is the ratio between a signal produced by an analyte and the quantity of analyte which produces the signal.

**↑increase production compared to control.**

**↓lower production compared to the control.**
